# Autism Associated Catatonia in Adolescence: The Critical Role of Environmental Structure in Relapse Prevention

**DOI:** 10.1192/bjo.2026.11791

**Published:** 2026-06-30

**Authors:** Hafijur Rahman, Mohammed Khan, Harshini Prasanna Kumar, Deniz Ezgi Yazici, Mohammed Fareeth Mohammed Farveez

**Affiliations:** Parkview Clinic Birmingham Womens and Childrens Trust, Birmingham, United Kingdom

## Abstract

**Aims:**

Autism Spectrum Disorder (ASD)-related catatonia presents a significant diagnostic and management challenge, due to the overlap between catatonic features and core autistic traits. Adolescents with ASD appear to be at increased risk of catatonic deterioration often triggered by psychosocial stressors and underlying neurobiological factors. This case describes a 16-year-old female adolescent whose catatonia was primarily driven by underlying autism, illustrating the crucial role of ASD-related mechanisms in both her deterioration and recurrence. Current literature review reveals a small number of documented adolescent catatonia cases indicating limited clinical and research consideration to this population.

**Methods:**

A multidisciplinary treatment approach integrating pharmacological, psychological, and environmental strategies was implemented. Pharmacological management included Lorazepam (1 mg BD) targeting motor symptoms, alongside Olanzapine (5 mg ON) and Sertraline (150mg OD) to address comorbid anxiety, emotional dysregulation, and behavioural rigidity. Regular physical health monitoring remained unremarkable throughout. Additionally, the adapted for children version of the Bush Francis catatonia scaled was utilised to measure symptoms. Psychological interventions focused on anxiety reduction, restoration of independence, and development of structured daily routines. Environmentally, a low-arousal setting was prioritised with consistent 1:1 support as part of Level 3 observations. This provided containment, predictability, relational stability and reduced sensory overload. Collaborative multidisciplinary working and graded Section 17 leave facilitated a smooth transition back to the community.

**Results:**

A clinical decline was observed when Lorazepam was tapered and observation levels reduced to Level 2 (15-minute checks). Catatonic features including behavioural “stuckness,” delayed motor initiation, increased ritualistic behaviour, and reduced self-care re-emerged. Marked improvement was observed following the reintroduction of 1:1 support as part of Level 3 observations and an increased Lorazepam dose. This clear temporal association emphasises that environmental structure and sustained relational support are as critical as pharmacological treatment.

**Conclusion:**

This case highlights the importance of recognising catatonia as a treatable manifestation of ASD. The clinical course established how ASD specific neurobiological vulnerabilities impacted to the initial deterioration and subsequent relapse. Recovery was dependent not only on benzodiazepine responsiveness but also on maintaining consistency, predictability, and therapeutic connection. A formulation driven, multidisciplinary approach balancing pharmacological, psychological, and environmental interventions is essential for sustaining improvement and preventing relapse in autistic adolescents with catatonia.

